# *Parvimonas micra* Bacteremia: A Rare Complication After Esophagogastroduodenoscopy for Upper Gastrointestinal Bleeding

**DOI:** 10.14309/crj.0000000000001378

**Published:** 2024-06-07

**Authors:** Hasan S. Raza, James S. Love, Adam E. Mikolajczyk

**Affiliations:** 1 Department of Medicine, University of Illinois, Chicago, IL; 2 Division of Gastroenterology and Hepatology, Department of Medicine, University of Illinois, Chicago, IL

**Keywords:** endoscopy, *Parvimonas micra*, bacteremia, gastrointestinal tract

## Abstract

*Parvimonas micra* bacteremia is rarely encountered in clinical practice. When it is, patients usually have underlying periodontal disease or colorectal carcinoma. To the best of our knowledge, this is the first case of *P. micra* bacteremia in a patient without the predisposing risk factors listed above. We postulate that this occurred because of translocation across an interrupted gut-blood barrier in the setting of an acute upper gastrointestinal bleed. We present this case to highlight the importance of identifying and treating *P. micra* bacteremia because it can prevent commonly encountered sequelae of untreated bacteremia and improve outcomes.

## INTRODUCTION

*Parvimonas micra* is an anaerobic Gram-positive coccus that is a part of the normal commensal flora of the gastrointestinal (GI) tract. *P. micra* bacteremia is rare and is usually seen in patients with underlying GI malignancies such as colorectal carcinoma (CRC) where *P. micra* is believed to contribute to tumorigenesis.^1^ Other predisposing factors include having an immunocompromised state, chronic renal insufficiency, periodontal disease, and perforated viscus.^2^ Although *P. micra* can cause infective endocarditis, bacterial meningitis, sepsis, and even death,^3–5^ there have only been a few cases of *P. micra* bacteremia in patients undergoing endoscopy in the absence of periodontal disease or underlying malignancy. One of these is in a patient who had good dental health and underwent an endoscopic retrograde cholangiopancreatography for choledocholithiasis.^6^ In this article, we present *P. micra* bacteremia in a patient without underlying periodontal disease or malignancy initially admitted for acute liver injury with a hospital course complicated by an acute upper GI bleed (GIB) secondary to severe Mallory-Weiss tear.

## CASE REPORT

**Figure 1.**
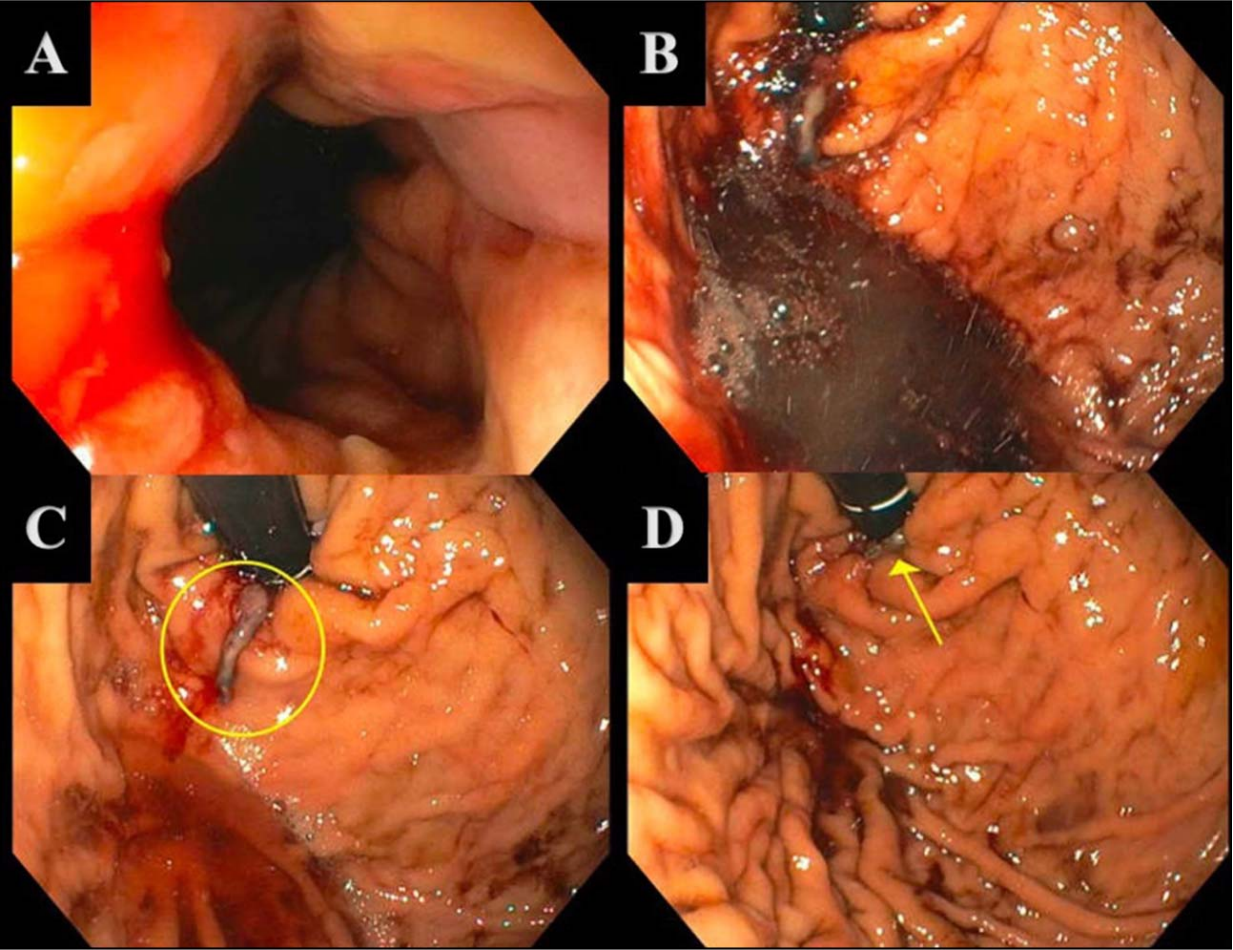
Gastroesophageal junction (GEJ) is visualized with active bleeding in (A) and blood in the gastric body in (B). In (C), the GEJ with LA grade (D) esophagitis and a Mallory-Weiss tear before clipping is visualized with a platelet plug circled. Arrow in (D) shows the clip with subsequent control of the bleed.

A 38-year-old man presented with diffuse abdominal pain and nonbloody, nonbilious emesis for 4 days after taking large, unknown quantities of acetaminophen and ibuprofen for management of pain after a physical altercation. Physical examination was notable for scleral icterus, diffuse abdominal tenderness, and normal dentition without evidence of periodontal disease. There was no asterixis with normal mentation. On admission, his serum creatinine was 11.86 mg/dL from baseline of 1.0 mg/dL. Liver function tests revealed aspartate transaminase of 77 U/L, alanine transaminase of 598 U/L, total bilirubin of 24.2 mg/dL, and direct bilirubin of 13.0 mg/dL. International normalized ratio was 1.8, and abdominal and pelvic computed tomography scans and right upper-quadrant ultrasound were unremarkable. Our patient was ultimately diagnosed with acute liver injury and toxic acute tubular necrosis secondary to acetaminophen and ibuprofen toxicities. He was treated with *n*-acetylcysteine and intravenous fluids with improvement in liver and kidney function. Despite improvement of creatinine, our patient had a persistently elevated blood urea nitrogen, and on day 4 of hospitalization, he developed melena and hemodynamic instability, concerning for an upper GIB. Urgent esophagogastroduodenoscopy (EGD) revealed active bleeding from a Mallory-Weiss tear at the gastroesophageal junction in the setting of Los Angeles grade D esophagitis with pooling of blood in the stomach (Figure 1). The Mallory-Weiss tear was treated with a hemostatic clip with subsequent control of the bleed (Figure 1). After the EGD, our patient's hemodynamics stabilized, and he requested a patient-directed discharge 1 day after the procedure. He returned after 3 days with lower extremity edema, pain, and fatigue. Two sets of blood cultures obtained in the emergency department eventually came back positive for *P. micra*. He was treated with ampicillin-sulbactam that was eventually transitioned to continuous infusion penicillin G for a 14-day course. Subsequent blood cultures remained negative. Chest x-ray and magnetic resonance imaging of the cervical spine did not reveal any signs of infection. Transthoracic echocardiogram did not reveal any findings, suggestive of infective endocarditis, and transesophageal echocardiogram was deferred to a later time should blood cultures have not cleared with antibiotics. Our patient was scheduled to be seen in outpatient hepatology clinic, although was unfortunately lost to follow-up.

## DISCUSSION

*P. micra* is a Gram-positive obligate anaerobe that is usually found in the GI and upper respiratory tracts of humans. Its ability to cause infection is associated with periodontal disease, alveolar or peritonsillar abscesses, chronic upper respiratory disease, such as chronic sinusitis or otitis media, and pulmonary pyogenic disease.^7^ *P. micra* has also been implicated in deeper infections such as those that occur around artificial joints. Interestingly, patients with a low socioeconomic status are more vulnerable to this infection, possibly related to barriers in obtaining routine preventive dental care.^8^ In addition, there is some evidence, which suggests that *P. micra* may be associated with CRC. In a study that investigated tumor colonization by *P. micra* and *Fusobacterium nucleatum*, there appeared to be an association between tumor colonization of *P. micra* and tumor resistance to therapies in CRC.^9,10^

Although there are previous case reports of *P. micra* in various clinical scenarios, this is the first documented case of *P. micra* bacteremia associated with GIB and endoscopic manipulation of the GI tract. Our patient did not have any of the predisposing conditions mentioned earlier and became bacteremic after instrumentation during the EGD. Given our patient's age and no family history of CRC, he had not received a screening colonoscopy. Abdominal computed tomography did not reveal any suspicious lesions. He was not due for any other routine cancer screening examinations.

In this case, there are several contributing etiologies to his bacteremia. Our patient had severe esophagitis with a Mallory-Weiss tear that allowed for disruption of the gut-blood barrier, which was then aggravated during endoscopic clipping. Fortunately, complications related to EGD are rare and occur in less than 2% of patients. The most common and serious complications are cardiopulmonary and are usually related to periprocedural sedation.^11^ Bacteremia is an uncommon complication and rarely reported from EGD with studies showing an overall frequency of 4.1%.^12^ The most common bacteria are *Escherichia coli*, *Salmonella* spp, and *Pseudomonas*, which are believed to occur from contaminated water sources causing ineffective endoscope reprocessing.^13^ In this patient with acute liver injury, there was an even greater risk of bacteremia because the liver plays a crucial role in clearing the blood of bacteria through the hepatic reticuloendothelial system and multiple defects in immune function (eg, opsonin and complement deficiencies, impaired neutrophil adherence, and Kupffer cell function) may have also been contributing.^14,15^

Regardless of route of transmission, *P. micra* bacteremia requires treatment to prevent common sequelae of bacteremia such as sepsis, endocarditis, and osteomyelitis. Since resistance to doxycycline and clindamycin has increased over time, it is important to note that *P. micra* remains susceptible to amoxicillin or metronidazole, which are the drugs of choice.^16^

## DISCLOSURES

Author contributions: All authors contributed to the case report. Manuscript preparation of the case and discussion was written by H.S. Raza. J.S. Love and A.E. Mikolajczyk edited the manuscript. All authors approved the final manuscript. H.S. Raza is the article guarantor.

Financial disclosure: None to report.

Previous presentation: An abstract of this case was accepted for presentation at the American College of Gastroenterology (ACG) Conference in Vancouver, Canada, and was presented on October 23, 2023.

Informed consent was obtained for this case report.
